# Waist Circumference and BMI in Relation to Serum High Sensitivity C-Reactive Protein (hs-CRP) in Cuban Americans With and Without Type 2 Diabetes

**DOI:** 10.3390/ijerph7030842

**Published:** 2010-03-08

**Authors:** Fatma G Huffman, Suzanne Whisner, Gustavo G Zarini, Subrata Nath

**Affiliations:** 1 Department of Dietetics & Nutrition, Florida International University, 11200 S.W. 8^th^ Street, Miami, FL 33199, USA; E-Mails: swhis001@fiu.edu (S.W.); gzarini@fiu.edu (G.G.Z.); 2 Department of Medicine, University of Texas Health Science Center at San Antonio, San Antonio, TX 78229, USA; E-Mail: nath@uthscsa.edu

**Keywords:** Type 2 diabetes, high-sensitivity C-reactive protein, waist circumference, body mass index

## Abstract

Relationship between high-sensitivity C-reactive protein (hs-CRP) and adiposity by diabetes status and gender in Cuban-Americans with and without type 2 diabetes (T2D) was studied. Adult subjects, 226 females, 129 males participated in a case control, single time point study. Subjects with T2D were older, had higher waist circumference (WC) and body mass index (BMI). WC and BMI were associated with ln hs-CRP (*P* < 0.001). An interaction with diabetes status was found for BMI (*P* = 0.037). Gender showed a strong relationship with ln hs-CRP (*P* < 0.001), which was moderated by diabetes status. Only males without diabetes exhibited a significant relationship for both WC and BMI with ln hs-CRP. In this sample of Cuban-Americans, WC and BMI had stronger associations with ln hs-CRP but not with diabetes status. Obesity prevention and controlling for CRP levels may be necessary to eliminate its contributions to develop diabetes and cardiovascular disease (CVD).

## 1. Introduction

As of 2007, approximately 24 million people in the United States had diabetes, with type 2 diabetes (T2D) accounting for 90–95% of all diabetes cases [1]. The incidence of diabetes is quickly multiplying, with an average of 1.6 million new cases diagnosed each year [2]. The most significant clinical consequence of T2D is mortality and morbidity from cardiovascular diseases (CVD), particularly coronary heart disease (CHD) and stroke [3]. Adults with diabetes are 2 to 4 times more likely to die from CHD and stroke than without diabetes, as almost 70% of deaths among those with diabetes are due to CHD or stroke [4,5].

Certain minority groups are at greater risk of diabetes than their Caucasian counterparts [6]. Specifically, Cuban-Americans between the ages of 45 and 74 have a 1.3 times higher prevalence of diabetes than similarly aged non-Hispanic whites according to the Hispanic Health and Nutrition Examination Survey (HHANES), a cross-sectional survey of the three major U.S. Hispanic populations conducted between 1982 and 1984 [7]. While HHANES provides data on the prevalence of diabetes for Cuban-Americans, it does not identify the risk factors and diabetes-related complications specific to this population. Furthermore, no data is available on the CHD risks in Cuban-Americans with T2D. Given that the prevalence of diabetes is about 16% among adult Cubans in the United States, it is of extreme importance that risk factors for CHD be identified in this rapidly proliferating, high-risk minority group [7].

In the past decade, it has become widely accepted that inflammation plays a key role in the pathogenesis of CVD. C-reactive protein (CRP), an acute phase protein and marker of chronic, low-grade inflammation, is a reliable predictor of CVD [8]. Data from prospective, epidemiologic studies revealed a significant association between CRP and future CHD risk in apparently healthy subjects [9]. Similarly, high plasma CRP has been shown to be an independent risk factor for CHD deaths in T2D [10].

CRP is made by the liver in response to inflammatory cytokines such as interleukin-6 (IL-6) and tumor necrosis factor-α (TNFα) [11]. Adipose tissue is a major source of these inflammatory cytokines [12]. Consequently, a strong positive association has been found between measures of obesity, such as waist circumference (WC) and body mass index (BMI), with CRP [13,14]. Moreover, while some studies have observed a relationship between T2D and higher CRP levels, it is unclear whether that relationship remains after adjustment for adiposity [15].

In addition to body fatness, CRP can be affected by age, smoking, medications, biochemical indices, dietary factors, lifestyle factors, genetics and race. Previous studies have shown that certain medications have an effect on CRP levels in the body [16–22]. Oral hypoglycemic medications such as metformin and rosiglitazone, singly or as combined treatment resulted in lower CRP levels in subjects with T2D [16,17]. Similarly antihypertensive medications such as beta blockers decreased overall CRP levels. Angiotensin-converting enzyme (ACE) inhibitors and angiotensin receptor blockers (ARB) lowered CRP only in subjects on monotherapy [18]. Selective serotonin reuptake inhibitor (SSRI) therapy in subjects with depressive disorder significantly decreased CRP levels whether or not the depressive condition was resolved [19]. Usage of aspirin and azithromycin by adults with unstable angina and cystic fibrosis respectively lowered CRP levels and improved quality of life [20,21]. Hormone replacement therapy (HRT) and raloxifene on the other hand had no effect on CRP levels in post menopausal women [22]. Dehghan *et al*. found significantly different serum CRP levels depending on CRP haplotypes, which remained after adjustment for obesity measures [23]. Furthermore, the distribution of CRP and the relationship between CRP and obesity vary according to race and gender, with certain minority groups having greater risks associated with CRP [24–26]. Still, few data regarding CRP, obesity and CVD risk have been generated for minority populations, and no information is available for CRP and related variables for Cuban-Americans, either healthy or dysmetabolic. Therefore, the purpose of this study was: (a) to examine differences in high-sensitivity C-reactive protein (hs-CRP) levels and body fatness by diabetes status, and; (b) to determine whether the relationship of hs-CRP with adiposity varies by diabetes status and gender in Cuban Americans.

## 2. Materials and Methods

### 2.1. Subjects

Data from a complete sample set of case control, single time point study of Cuban-Americans with and without T2D was used in the present study. Recruitment of participants was conducted in alternative phases of potential subjects with and without T2D. During a 1-year period, approximately 10,000 letters outlining the study were mailed to subjects with and without diabetes ages 30 years or older. Letters were sent in English and Spanish, and included an invitation flyer to which interested participants could respond. The participants were initially recruited by random selection (every tenth address) from a randomly generated mailing list. The list of addresses was purchased from Knowledge Base Marketing Inc., Richardson, TX 75081. This company provided mailing list of Cuban Americans from Miami-Dade and Broward Counties, Florida. Three percent (n = 300) of the letters were returned due to unknown addresses. From the remaining delivered mail, 4% (n = 388) responded. Interested participants were initially interviewed on the phone, at which time the study purpose was explained and age and gender of the responders were determined. To ascertain T2D status, each participant was asked for the age of diagnosis and initial treatment modalities. Inclusion criteria for the T2D group were as follows: (1) Cuban-American, (2) self-reported T2D, (3) aged >30 years, male or female, (4) not pregnant or lactating, (5) no thyroid disorders, and (6) no major psychiatric disorders. Inclusion criteria for group without diabetes met the same criteria as subjects with T2D except they were free from any type of diabetes. Eighteen subjects did not qualify for the study; for not being Cuban-American (n = 2), age (n = 9) or having other chronic illness (n = 7). If a subject was determined to be eligible, then their participation was requested at the Human Nutrition Laboratory at Florida International University (FIU). Participants were instructed to refrain from smoking, consuming any food or beverages except water, and any unusual exercise for at least eight hours prior to their blood collection. The approved Institutional Review Board informed consent was presented to eligible participants in Spanish or English based on their preference and signatures were obtained prior to the commencement of the study. Three subjects were excluded due to missing values needed for analysis. We classified T2D based on the self-report of the participants and we were able to verify subjects’ own account by blood tests. Those who reported not having T2D (n = 7) were classified as having T2D based on fasting plasma glucose (FPG) and HbA_1c_ analysis. Individuals were classified as having T2D based on the same criteria that was applied to those reported having T2D and those who did not. HbA_1c_ is an overtime measure of blood glucose. In individuals who reported not having T2D measuring HbA_1c_ was a good indicator of FPG having been high at least 2−3 months prior to this measure. These subjects were given their laboratory results and referred to their physicians.

### 2.2. Procedure

Subjects were asked to fill out standard questionnaires on site. Trained interviewers who were bilingual in English and Spanish were present to administer the questionnaires. Through administration of questionnaires, information was obtained on subjects’ socio-demographic data, smoking history and status, diabetes status, diabetes care (for T2D only), anthropometric measurements and nutritional variables. Height and weight were measured using a SECA balance scale (Seca Corp, Columbia, MD). BMI was calculated as weight in kg/height in m^2^. Waist circumference (WC) to the nearest 0.1 cm was measured horizontally with a non-stretchable measuring tape placed midway between the 12^th^ rib and iliac crest at minimal respiration to determine central obesity [27]. A 20 ml venous blood was collected from each subject after an overnight fast (at least 8 hours) by a certified phlebotomist using standard laboratory techniques. Blood samples were collected into 2 tubes: one containing K2EDTA to analyze HbA_1c_ and the second a vaccutainer Serum Separator Tube (SST) for analysis of glucose. After complete coagulation (30–45 minutes), the SST was centrifuged at 2,500 RPM for 30 minutes. The serum was transferred from the spun SST into 3 labeled plastic tubes: the first tube was used for glucose analysis, and the second tube was stored at −70 °C to be used later for hs-CRP. Glucose levels were measured by hexokinase enzymatic methods by Laboratory Corporation of America, Miami, FL (LabCorp®). Hs-CRP was analyzed at the Vascular Disease Intervention and Research Laboratory, Edmond, OK using Immulite method. The Immulite assay is a 2-site chemiluminescent enzyme immunometric assay with one monoclonal and one polyclonal anti-CRP antibody. A 1:100 manual dilution of the antibody provides a measurable range of 0.1–500 mg/L [28].

### 2.3. Statistical Analysis

SPSS program (Version 15.0, Chicago, IL) was used for statistical analysis and statistical significance was accepted at *P* < 0.05. Data not normally distributed were natural log (ln) transformed to approach normality and outliers were excluded from analysis. Descriptive statistics were generated for all variables. Differences in mean values between subjects with and without T2D were assessed using the unpaired Student’s *t*-test for numerical values and chi-square test for categorical variables to test the first aim. Pearson’s correlations and Multiple Linear Regression (MLR) was conducted to test the second aim, using ln hs-CRP as a dependent variable and WC or BMI as independent variables stratified by gender and diabetes status. Age, smoking, non-steroidal anti-inflammatory drugs (NSAIDS), cholesterol medications, hypertension medications, depression medications and diabetes medications were included as controlling variables.

## 3. Results

The present study analyzed 355 subjects, 226 females and 129 males. Characteristics of the study population are shown (Table 1). Subjects with T2D were older than subjects without diabetes (65.3 ± 12.1y *vs.* 62.5 ± 11.4y, respectively). Also, subjects with diabetes had significantly higher body adiposity with a greater BMI (31.6 ± 6.6 kg/m^2^ *vs.* 30.1 ± 5.2 kg/m^2^) and WC (105.7 ± 14.7 cm *vs.* 100.3 ± 12.4 cm) than subjects without diabetes. Non-steroidal anti-inflammatory drug (NSAID) use was significantly higher in subjects without diabetes (50% *vs.* 31%, respectively). Subjects with T2D had significantly higher fasting plasma glucose (FPG) and HbA_1c_ than those without (146.1 ± 65.7 *vs.* 98.0 ± 17.9 and 7.5 ± 1.5 *vs.* 5.9 ± 0.6, respectively). Hs-CRP levels were skewed; therefore, values were natural log (ln) transformed. Ln hs-CRP was not significantly different for subjects with T2D than without diabetes. Similarly, no difference was detected between the two groups for gender, active smoking, cholesterol medication use, hypertension medication use or depression medication use.

After adjustment for age, smoking, diabetes status and medication use (NSAID, cholesterol, hypertension and depression medications), MLR revealed that WC, BMI, and gender were all significantly associated with ln hs-CRP (*P* < 0.05) (Tables 2 and 3).

Both WC and BMI showed a strong positive relationship with ln hs-CRP (*P* < 0.001). No significant interaction was revealed for WC with diabetes status (*P* = 0.065) but an interaction between BMI and diabetes status was found (*P* < 0.05). The slope for the relationship between BMI and ln hs-CRP was stronger for persons without diabetes than with diabetes (B = 0.099 and B = 0.055, respectively), but both slopes were significantly different than zero (*P* < 0.001).

Since gender was shown to be related to ln hs-CRP, further analysis was layered by gender and diabetes status (Table 4). No difference was found between males with diabetes and without diabetes for age, BMI, WC and ln hs-CRP. Females, however, varied significantly for age, BMI and WC. Specifically, females with diabetes were older and had greater BMIs and WCs than without diabetes (*P* < 0.05 for all variables).

Pearson’s correlations showed that ln hs-CRP was significantly correlated with WC (r = 0.470, p = 0.001 and r = 0.406, p = 0.001) and BMI (r = 0.387, p = 0.001 and r = 0.395, p = 0.001) among females with and without diabetes. However, ln hs-CRP was significantly correlated with WC (r = 0.563, p = 0.001) and BMI (r = 0.548, p = 0.001) only among males without diabetes. MLR analysis showed that diabetes status did not affect the relationship between WC and BMI with ln hs-CRP in females. A significant difference moderated by diabetes status was found in males associated with both WC (P = 0.004) and BMI (P = 0.009) with ln hs-CRP (Table 5). When controlling for age, smoking, and medications, males without diabetes exhibited a stronger and significant relationship between WC and ln hs-CRP compared with males with diabetes (B = 0.048, *P* < 0.001 and B = 0.004, *P* = 0.712, respectively). A similar association was revealed for BMI and ln hs-CRP, with males without diabetes showing a stronger relationship (B = 0.127, *P* < 0.001) than with diabetes (B = 0.020, *P* = 0.458).

## 4. Discussion

The present study confirms previous observations regarding the systemic inflammation associated with greater adiposity as measured by waist circumference and BMI [13,14,24,26,29]. Hs-CRP did not vary by to diabetes status, but was strongly associated with both waist circumference and BMI. These results are congruent with findings from other investigations [15,24]. Efstratiadis *et al*. discovered that after controlling for BMI and various CRP confounders, diabetes was not associated with CRP [30]. Still, prospective, epidemiological studies have shown high CRP to be a major contributor to the risk of T2D, independent of measures of body adiposity [23].

Data from HHANES revealed that among Cuban American individuals 18 years or older the prevalence of obesity was 15% for females and 9% for males [31]. The National Health Interview Survey, Sample Adult File (NHIS-SAF) and HHANES indicated that the prevalence of obesity and diabetes varies among Hispanics [32]. Mexican Americans had a higher prevalence of obesity (25.3%) and diabetes (23.9%) compared to Cuban Americans (20.2% and 15.8% respectively) [7,32]. In contrast, according to the National Vital Statistics System (NCHS) diabetes related deaths was higher among Cuban Americans (44%) compared Puerto Ricans (39%) and Mexican Americans (37%) [33].

Subjects with diabetes were found to be older and more obese than subjects without diabetes in this group of Cuban-Americans, which is in agreement with results from multi-ethnic populations [30,34,35]. However, it is important to consider the influence of race and ethnicity when interpreting the results of the present study. Patel *et al*. examined the relation of CRP with waist circumference and other correlates of the metabolic syndrome [25]. Racial differences were found for the association of waist circumference and CRP, highlighting the need for a better understanding of the significance of CRP and clinical relevance of values for various ethnicities and races. Indeed, the majority of the current literature on CRP has been generated from mostly Caucasian populations. To our knowledge, this study was the first to investigate the relationship of obesity and diabetes with hs-CRP in a Cuban-American population. Thus, the present analysis is largely exploratory in nature, but serves to add clinical insight on the inflammatory burden of non-white populations.

Our investigation also revealed the possibility of a gender influence on hs-CRP and body fatness. Females exhibited a strong association for both waist circumference and BMI with hs-CRP, regardless of diabetes status. Males, however, differed according to the presence or absence of diabetes. For men without diabetes the association between waist circumference and BMI with hs-CRP was much stronger than for men with diabetes. Namely, for the same waist circumference or BMI, men without diabetes had higher hs-CRP levels than men with diabetes. Still, no such relation was found in the literature or in our study for females, despite previous reports to the contrary [26,36].

In general, body fatness had a greater affect on hs-CRP for subjects without diabetes than subjects with diabetes, which was an unexpected result. This finding may be partially explained by the influence of medications for diabetes. Since the analysis was layered by diabetes status, the potential confounding of medications taken only by subjects with diabetes could not be controlled for. Insulin, for example, has been shown to act as an anti-inflammatory modulator and some oral hypoglycemic agents (OHAs) can affect hs-CRP levels [37,38]. Thus, the use of insulin or OHAs could have artificially lowered hs-CRP levels in subjects with diabetes, making a difference between the groups difficult to detect.

The single time point design of this study is a limitation of our research. Specifically, the temporal relationship between body fatness, T2D, inflammation or cardiovascular risk cannot be revealed. Furthermore, some bias may be introduced through the use of a semi-convenience sample. Hence, our findings cannot be generalized to the greater Cuban-American community. Another limitation to this study was the ratio of males to females, in that the study population included almost two females for every male. However, the value of the current research was augmented by the inclusion of both males and females, and by the resulting gender difference observed in this previously understudied population. Overall, our study has important clinical and public health implications. Cuban-Americans are the fastest growing Hispanic group at high-risk for both T2D and cardiovascular disease. Yet, little research has been conducted on Cuban-Americans separate from a general Hispanic population. This exploratory study highlights a possible role for gender and an influence of ethnicity. As the basis for future research, these findings may contribute to the development of ethnicity-based health interventions for chronic diseases.

## Figures and Tables

**Table 1.** Characteristics of study population by diabetic status.

*Variables*	*Without Diabetes (n = 178)*	*With Diabetes (n = 177)*	*P-value*†
Age (years)	62.5 ± 11.4	65.3 ± 12.1	0.023
Gender (female %)	65	60	0.322
BMI (kg/m^2^)	30.1 ± 5.2	31.6 ± 6.6	0.022
WC (cm)	100.3 ± 12.4	105.7 ± 14.7	0.001
Ln hs-CRP (mg/L)	1.03 ± 1.18	1.04 ± 1.23	0.901
Active smokers (%)	18	15	0.667
NSAID (yes %)	50	31	0.001
Cholesterol meds (yes %)	18	21	0.510
Hypertension meds (yes %)	47	53	0.289
Depression meds (yes %)	14	11	0.516
Diabetes meds (yes %)	0	82.5	0.001
FPG (mg/dl)	98.0 ± 17.9	146.1 ± 65.7	0.001
HbA_1c_ (%)	5.9 ± 0.6	7.5 ± 1.5	0.001

Quantitative variables are expressed as mean ± standard deviation (SD).

† Evaluated by t-test for numerical variables and chi-square test for categorical variables; *P* < 0.05 considered significant. Abbreviations: BMI, body mass index; WC, waist circumference; NSAID, non-steroidal antiinflammatory drug; meds, medications, FPG, fasting plasma glucose, HbA_1c_, glycated hemoglobin. FPG (n = 173 without diabetes and n = 174 with diabetes), HbA_1c_ (n = 177 without diabetes and n = 174 with diabetes).

**Table 2.** Relationship of waist circumference and covariates with ln hs-CRP layered by diabetes status.

*Parameter*†	*B*	*SE*	*T*	*P-value*‡
WC	0.043	0.007	6.183	<0.001
Diabetes_Status*WC	−0.017	0.009	−1.850	0.065
Gender	−0.333	0.129	−2.589	0.010

† Other covariates appearing in the Multiple Linear Regression (MLR) are age, smoking, NSAID, cholesterol medication, hypertension medication, depression medication; diabetes medication.

‡ *P* < 0.05 is considered significant. Abbreviations: WC, waist circumference; SE, standard error; B, slope parameter; Diabetes_Status*WC, variable testing for interaction between diabetes status and waist circumference; Diabetes_Status = 1 with diabetes; 0 = without diabetes; Gender = 1 male, 0 = female.

**Table 3.** Relationship of body mass index and covariates with ln hs-CRP by diabetes status.

*Parameter*†	*B*	*SE*	*T*	*P-value*‡
BMI				
Without Diabetes	0.099	656	−1.95	<0.001
With Diabetes	0.055	0.013	4.09	<0.001
Diabetes_Status *BMI	−0.044	0.021	−2.089	0.037

† Other covariates appearing in the Multiple Linear Regression (MLR) are age, gender, smoking, NSAID, cholesterol medication, hypertension medication, depression medication; diabetes medication.

‡ *P* < 0.05 is considered significant. Abbreviations: BMI, body mass index; SE, standard error; B, slope parameter; Diabetes_Status*BMI, variable testing for interaction between diabetes status and body mass index.

**Table 4.** Characteristics of subjects grouped by gender and diabetes status.

	*Males*		
*Parameter*	*Without diabetes (n = 60)*	*With Diabetes (n = 69)*	*P-value*†

Age (years)	62.6 ± 11.4	63.8 ± 11.3	0.551
BMI (kg/m^2^)	29.4 ± 5.1	30.3 ± 5.3	0.325
WC (cm)	102.9 ± 13.3	106.6 ± 13.7	0.123
Ln hs-CRP	0.81 ± 1.18	0.95 ± 1.21	0.518
	*Females*		
*Parameter*	*Without diabetes (n = 118)*	*With Diabetes (n = 108)*	*P-value*†

Age (years)	65.6 ± 11.3	66.11 ± 12.4	0.016
BMI (kg/m^2^)	30.5 ± 5.2	32.4 ± 7.1	0.024
WC (cm)	98.9 ± 11.8	105.1 ± 15.3	0.001
Ln hs-CRP	1.1 ± 1.2	1.1 ± 1.3	0.838

Values are expressed as mean ± standard deviation (SD).

† Evaluated by t-test; *P* < 0.05 considered significant. Abbreviations: BMI, body mass index; WC, waist circumference.

**Table 5.** Relationship of waist circumference and BMI with ln hs-CRP by diabetes status for male subjects.

*Diabetes Status*	*Waist Circumference*			*P-value*†
	*B*	*SE*	*t*	

Without diabetes	0.048	0.011	4.41	<0.001
With Diabetes	0.004	0.010	0.37	0.712
	*BMI*			*P-value*†
	*B*	*SE*	*t*	

Without diabetes	0.127	0.029	4.35	<0.001
With Diabetes	0.020	0.027	0.74	0.458

Other covariates appearing in the Multiple Linear Regression (MLR) are age, smoking, NSAID, cholesterol medication, hypertension medication, depression medication; diabetes medication.

† *P* < 0.05 is considered significant. Abbreviations: SE, standard error; BMI, body mass index; B, slope parameter.

## References

[1] American Diabetes Association Total prevalence of diabetes and pre-diabetes Available online: http://www.diabetes.org/diabetes-statistics/prevalence.jsp (accessed on March 16, 2009).

[2] Center for Disease Control CDC Diabetes Fact Sheet 2007 Available online: http://www.cdc.gov/diabetes/pubs/pdf/ndfs_2007.pdf (accessed on March 16, 2009).

[3] Schulze MB Rimm EB Li T Rifai N Stampfer MJ Frank B C-reactive protein and incident cardiovascular events among men with diabetes Diabetes Care 2004 27 889 893 15047644 10.2337/diacare.27.4.889

[4] Howard BV Rodriguez BL Bennett PH Harris MI Hammam R Kuller LH Pearson TA Wylie-Rosett J Prevention Conference VI: diabetes and cardiovascular disease: writing group I: epidemiology Circulation 2002 105 132 137 11994263 10.1161/01.cir.0000013953.41667.09

[5] Rijelijkhutzen JM Nijpels G Heine RJ Bouter LM Stehouwer CDA Dekker JM High risk of cardiovascular mortality in individuals with impaired fasting glucose is explained by conversion to diabetes Diabetes Care 2007 30 332 336 17259503 10.2337/dc06-1238

[6] Hummer RA Rogers RG Amir SH Forbes D Frisbie WP Adult mortality differentials among Hispanic subgroups and non-Hispanic whites Soc. Sci. Q 2000 81 459 476 17879490

[7] Flegal KM Ezzati TM Harris MI Haynes SG Juarez RZ Knowler WC Perez-Stable EJ Stern MP Prevalence of diabetes in Mexican Americans, Cubans, and Puerto Ricans from the Hispanic Health and Examination Survey, 1982–1984 Diabetes Care 1991 14 628 638 1914812 10.2337/diacare.14.7.628

[8] Ridker PM Hennekens CH Buring JE Rifai N C-reactive protein and other markers of inflammation in the prediction of cardiovascular disease in women N. Engl. J. Med 2000 342 836 843 10733371 10.1056/NEJM200003233421202

[9] Danesh J Whincup P Walker M Lennon L Thomson A Appleby P Gallimore RJ Pepys MB Low grade inflammation and coronary heart disease: prospective study and updated meta-analyses BMJ 2000 321 199 204 10903648 10.1136/bmj.321.7255.199 PMC27435

[10] Soinio M Marniemi M Laasko S Lehto J Ronnemaa T High sensitivity C-reactive protein and coronary heart disease mortality in patients with type 2 diabetes: a 7 year follow up study Diabetes Care 2006 29 329 333 16443882 10.2337/diacare.29.02.06.dc05-1700

[11] Bullo M Garcia-Lorda P Megias I Salas-Salvado J Systemic inflammation, adipose tissue tumor necrosis factor, and leptin expression Obes. Res 2003 85 3338 3342 10.1038/oby.2003.74 12690081

[12] Yudkin JS Stehouwer CDA Emeis JJ Coppack SW C-reactive protein in healthy subjects: Associations with obesity, insulin resistance and endothelial dysfunction: a potential role for cytokines originating from adipose tissue Int. J. Obes 1999 19 972 978 10.1161/01.atv.19.4.972 10195925

[13] Santos AC Lopes C Guimaraes JT Barros H Central obesity as a major determinant of increased high-sensitivity C-reactive protein in metabolic syndrome Int. J. Obes 2005 29 1452 1456 10.1038/sj.ijo.0803035 16077717

[14] Shemesh T Rowley KG Jenkins A Brimblecombe J Best JD O’Dea K Differential association of C-reactive protein with adiposity in men and women in an Aboriginal community in northeast Arnhem Land of Australia Int. J. Obes 2007 31 103 108 10.1038/sj.ijo.0803350 16682979

[15] Onat A Can G Hergenc G Serum C-reactive protein is an independent risk factor predicting cardiometabolic risk Metab. Clin. Experim 2008 57 207 214 10.1016/j.metabol.2007.09.002 18191050

[16] Farah R Shurtz-Swirski R Lapin O Intensification of oxidative stress and inflammation in type 2 diabetes despite antihyperglycemic treatment Cardiovasc. Diabetol 2008 22 7 20 10.1186/1475-2840-7-20 PMC2441608 18570678

[17] Stewart MW Cirkel DT Furuseth K Donaldson J Biswas N Starkie MG Phenekos C Hamann A Effect of metformin plus roziglitazone compared with metformin alone on glycaemic control in well-controlled Type 2 diabetes Diabet. Med 2006 23 1069 1078 16978370 10.1111/j.1464-5491.2006.01942.x

[18] Palmas W Ma S Psaty B Goff DC Darwin C Barr RG Antihypertensive Medications and C-Reactive Protein in the Multi-Ethnic Study of Atherosclerosis Am. J. Hypertens 2007 20 233 241 17324732 10.1016/j.amjhyper.2006.08.006

[19] O’brien SM Scott LV Dinan TG Antidepressant therapy and C-reactive protein levels Br. J. Psychiatry 2006 188 449 452 16648531 10.1192/bjp.bp.105.011015

[20] Kennon S Price CP Mills PG Ranjadayalan K Cooper J Clarke H Timmis AD The effect of aspirin on C-reactive protein as a marker of risk in unstable angina J. Am. Coll. Cardiol 2001 37 1266 1270 11300433 10.1016/s0735-1097(01)01130-5

[21] Wolter J Seeney S Bell S Bowler S Masel P McCormack J Effect of long term treatment with azithromycin on disease parameters in cystic fibrosis: a randomised trial Thorax 2002 57 212 216 11867823 10.1136/thorax.57.3.212 PMC1746273

[22] Walsh BW Paul S Wild RA Dean RA Tracy RP Cox DA Anderson PW The effects of hormone replacement therapy and raloxifene on C-reactive protein and homocysteine in healthy postmenopausal women: A Randomized, Controlled Trial J. Clin. Endocrinol. Metab 2000 85 214 218 10634389 10.1210/jcem.85.1.6326

[23] Dehghan A Kardys I De Maat MPM Uitterlinden AG Sijbrands EJG Bootsma AH Stijnen T Hofman A Schram MT Witteman JCM Genetic variation, C-reactive protein levels and incidence of diabetes Diabetes 2007 56 872 878 17327459 10.2337/db06-0922

[24] Onat A Sari I Hergenc G Yazici M Uyarel H Can G Sansoy V Predictors of abdominal obesity and high susceptibility of cardiometabolic risk to its increments among Turkish women: a prospective population-based study Metab. Clin. Experim 2007 56 348 356 10.1016/j.metabol.2006.10.016 17292723

[25] Patel DA Srinivasan SR Xu JH Li S Chen W Berenson GS Distribution and metabolic syndrome correlates of plasma C-reactive protein in biracial (black-white) younger adults: the Bogalusa Heart Study Metab. Clin. Experim 2006 55 699 705 10.1016/j.metabol.2005.07.015 16713426

[26] Florez H Castillo-Florez S Mendez A Casanova-Romero P Larreal-Urdaneta C Lee D Goldberg R C-reactive protein is elevated in obese patients with the metabolic syndrome Diaetes Res. Clin. Pract 2006 71 92 100 10.1016/j.diabres.2005.05.003 16002176

[27] Callaway CW Chumlea WC Bouchard C Himes JH Lohman TG Martin AD Mitchell CD Mueller WH Roche AF Seefeldt VD Circumferences Lohman TG Roche AF Martorell R Anthropometric standardization reference manual Human Kinetics Champaign, IL, USA 1988 39 54

[28] Roberts WL Sedrick R Moulton L Spencer A Rifai N Evaluation of four automated high-sensitivity C-reactive protein methods: implications for clinical and epidemiological; applications Clin. Chem 2000 46 461 468 10759469

[29] Muscari A Bastagli L Poggiopollini G Tomassetti V Massarelli G Cappelletti O Plate L Boni P Puddu P Different associations of C-reactive protein, fibrinogen and C3 with traditional risk factors in middle-aged men Inter. J. Cardiol 2002 83 63 67 10.1016/s0167-5273(02)00017-7 11959386

[30] Efstratiadis G Tsiaousis G Athyros VS Karagianni D Pavlitou-Tsiontsi A Giannakou-Darda A Manes C Total serum insulin-like growth factor-1 and C-reactive protein in metabolic syndrome with or without diabetes Angiology 2006 57 303 310 16703190 10.1177/000331970605700306

[31] Pawson IG Martorell R Mendoza FE Prevalence of overweight and obesity in US Hispanic populations Am. J. Clin. Nutr 1991 53 1522S 1528S 2031482 10.1093/ajcn/53.6.1522S

[32] Denney JT Krueger PM Rogers RG Boardman JD Race/ethnic and sex differentials in body mass among US adults Ethn. Dis 2004 14 389 398 15328941

[33] Smith CA Barnett E Diabetes-related mortality among Mexican Americans, Puerto Ricans, and Cuban Americans in the United States Rev. Panam. Salud Publica 2005 18 381 387 16536924 10.1590/s1020-49892005001000001

[34] Haffner SM The metabolic syndrome: inflammation, diabetes mellitus, and cardiovascular disease Am. J. Cardiol 2006 96 3A 11A 10.1016/j.amjcard.2005.11.010 16442931

[35] Bartels DW Davidson MH Gong WC Type 2 diabetes and cardiovascular disease: reducing the risk J. Manag. Care Pharm 2007 13 S2 S15 17330977 10.18553/jmcp.2007.13.s2-a.1 PMC10437557

[36] Williams K Tchernof A Hunt KJ Wagenknecht LE Haffner SM Sniderman AD Diabetes, abdominal adiposity, and atherogenic dyslipoproteinemia in women compared with men Diabetes 2008 57 3289 3296 18809621 10.2337/db08-0787 PMC2584135

[37] Dandona T Chaudhuri A Ghanim H Mohanty P Insulin as an anti-inflammatory and antiatherogenic modulator J. Amer. Coll. Cardiol 2009 53 S14 S20 19179212 10.1016/j.jacc.2008.10.038

[38] Huang CC Treatment targets for diabetic patients on peritoneal dialysis: any evidence? Perit. Dial. Int 2007 27 176 179 17556300

